# Investigation of protein family relationships with deep learning

**DOI:** 10.1093/bioadv/vbae132

**Published:** 2024-09-18

**Authors:** Irina Ponamareva, Antonina Andreeva, Maxwell L Bileschi, Lucy Colwell, Alex Bateman

**Affiliations:** European Molecular Biology Laboratory, European Bioinformatics Institute (EMBL-EBI), Wellcome Genome Campus, Hinxton, Cambridgeshire CB10 1SD, United Kingdom; Department of Chemistry, University of Cambridge, Cambridge CB2 1EW, United Kingdom; European Molecular Biology Laboratory, European Bioinformatics Institute (EMBL-EBI), Wellcome Genome Campus, Hinxton, Cambridgeshire CB10 1SD, United Kingdom; Google Research, Cambridge, MA 02142, United States; Department of Chemistry, University of Cambridge, Cambridge CB2 1EW, United Kingdom; Google Research, Cambridge, MA 02142, United States; European Molecular Biology Laboratory, European Bioinformatics Institute (EMBL-EBI), Wellcome Genome Campus, Hinxton, Cambridgeshire CB10 1SD, United Kingdom

## Abstract

**Motivation:**

In this article, we propose a method for finding similarities between Pfam families based on the pre-trained neural network ProtENN2. We use the model ProtENN2 per-residue embeddings to produce new high-dimensional per-family embeddings and develop an approach for calculating inter-family similarity scores based on these embeddings, and evaluate its predictions using structure comparison.

**Results:**

We apply our method to Pfam annotation by refining clan membership for Pfam families, suggesting both new members of existing clans and potential new clans for future Pfam releases. We investigate some of the failure modes of our approach, which suggests directions for future improvements. Our method is relatively simple with few parameters and could be applied to other protein family classification models. Overall, our work suggests potential benefits of employing deep learning for improving our understanding of protein family relationships and functions of previously uncharacterized families.

**Availability and implementation:**

github.com/iponamareva/ProtCNNSim, 10.5281/zenodo.10091909.

## 1 Introduction

The classification of protein sequences into domain families is an important topic in *Bioinformatics* leading to highly cited tools and data resources (Matelska *et al.* 2017, Mistry *et al.* 2021). However, we are still quite a long way from having a full understanding and knowledge of all protein domain families. There have been significant recent advances in the field, such as the availability of millions of accurate structural models (Varadi and Velankar 2022, Lin *et al.* 2023), and fast structural aligners such as foldseek (van Kempen *et al.* 2023), which have improved our ability to accurately identify protein domain boundaries and find relationships between them. Deep learning methods have also been applied to identify and expand the coverage of protein sequence annotation. The first version of the ProtENN method (Bileschi *et al.* 2022) increased the sequence coverage of Pfam (the fraction of sequences with at least one domain match) by 9.5% and a new version of that method (ProtENN2, described briefly below) increases the residue coverage of Pfam by a further 12% (Bileschi *et al.* manuscript in preparation, https://zenodo.org/records/11187183).

An important task in the classification of protein families is the identification of homology relationships between families. Discovering protein family homology aids our understanding of protein evolution and protein function because related families often have related functions. A clear case where finding relationships between families may be especially useful is when characterizing domains of unknown function (DUFs) (Bateman *et al.* 2010). This can be done at the level of sequence (Matelska *et al.* 2017) or structure (Holm *et al.* 2023) Despite decades of work, in many cases, relationships between different protein families and the functions of many families are still unclear. Pfam release 35.0 contains 4795 families with a DUF designation and only 7769 of 19 632 families belong to a clan. We could improve the annotation further with additional accurate knowledge of inter-family relationships.

In this work, we develop a method that uses the ProtENN2 sequence embeddings to derive family representations and thereby predict protein family similarity. ProtENN2 is an ensemble of ResNet-style convolutional models that provide Pfam domain annotations for each residue of an input protein sequence (Bileschi *et al.*, manuscript in preparation, https://zenodo.org/records/11187183). This model is a step forward from the per-domain ProtENN model (Bileschi *et al.* 2022), which produces a single representation for each labelled Pfam domain instance that occurs within a sequence. We suggest that features learned by neural network models, such as ProtENN2, could in principle go beyond relying on sequence similarity and discover other features that improve the accuracy of similarity prediction. One such feature could be the domain architecture of proteins, which can be used by the model due to its global nature. For example, a weak match between two domain families might be strengthened by sharing a common accessory C-terminal domain.

## 2 Methods

To approach the problem of finding relationships between protein families, we create family-specific high-dimensional vector representations. We do this using embeddings produced by the ProtENN2 model, which was trained to address the problem of assigning Pfam domains to protein sequence regions. The ProtENN2 model also will predict Clan labels, but we do not use these predictions in this application. The embeddings are then aggregated for each family, and a similarity metric on these embeddings is introduced.

### 2.1 ProtENN2

ProtENN was introduced in 2019 to identify Pfam families for input protein domain sequences (Bileschi *et al.* 2022). This method is based on an ensemble of ResNet-style convolutional models that calculate a single embedding for each domain sequence. Although the method annotates pre-cut domain sequences with high accuracy, it only provides a single label per input sequence and thus is not able to annotate a complete protein sequence that may contain multiple Pfam domains. The detailed architecture of the model is described in Bileschi *et al.* (2022). ProtENN2 further develops the ProtENN framework, enabling a Pfam family and/or clan annotation to be predicted for every residue of an input protein sequence. The ProtENN2 model architecture is the same as the ProtENN model architecture, with a slightly modified output layer, which provides per-residue probabilities instead of per-sequence ones. This model is a step forward from the per-domain model (Bileschi *et al.* 2022) because it enables multiple domain labels to be predicted per sequence and moreover the domain boundaries are directly predicted by the model.

**Figure 1.**
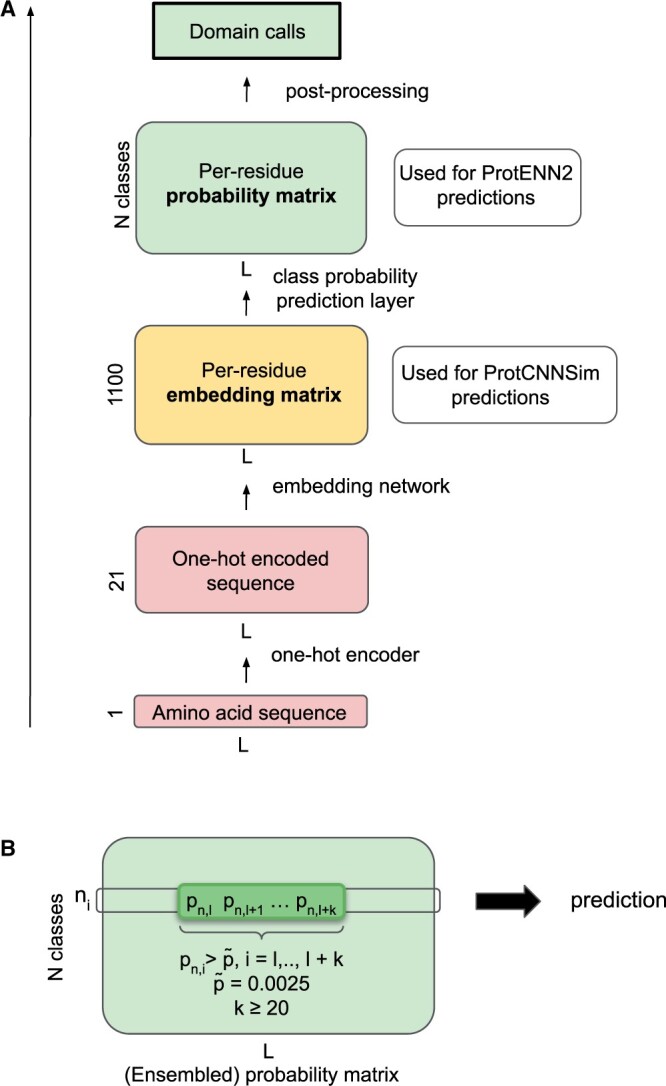
Schematic diagram of the ProtENN2 model architecture. (A) Diagram showing the architecture of a single model from the ensemble. The embedding matrix precedes the class probability prediction layer and is generated by passing the sequence through the input network and convolutional blocks. (B) In the probability matrix, domain calls (ProtENN2 predictions) are made by selecting continuous spans of positions that pass both length and probability thresholds.

The ProtENN2 method uses an ensemble of 31 individual convolutional models. Each member of the ProtENN2 ensemble consists of five residual layers. Input sequences are fed to the model as sequences of amino acid characters. Each input sequence is one-hot encoded before the residual blocks, yielding a 21-channel vector representation for each position. After passing through the convolutional layers, the model generates per-residue embeddings of size 1100, which are then passed to the fully connected classification layer. The output produced by the model is a class probability vector for each amino acid residue of a sequence, where classes represent Pfam families and/or clans (Fig. 1A). The final prediction is made by averaging the probability predictions for each position and each class among all the models in the ensemble. As discussed in Bileschi *et al.* (manuscript in preparation, https://zenodo.org/records/11187183), clan membership is used as supervision during training, however, in this work, we only consider the 19 632 Pfam family outputs of the model.

We use ProtENN2 models trained on Pfam version 35.0, which contains 19 632 families. The results of running this model are called Pfam-N and are made available with the Pfam release files and via the InterPro website. ProtENN2 provides accurate predictions of Pfam families and was adopted to extend Pfam coverage significantly. Additionally, the previous generation of the model, the per-sequence ProtENN model, was shown to produce embeddings that have useful properties for clustering and clan-level function prediction (Bileschi *et al.* 2022, Sanderson *et al.* 2023). This suggests that embeddings that serve as inputs to the classification layer might have properties or features that can help to identify family similarity.

### 2.2 Family embeddings and similarity scores

We use the per-residue embeddings produced by the last convolutional layer of a single ProtENN2 model to build family-specific embeddings, which are then used to evaluate family similarity. To obtain predictions, we use the Pfamseq 35 database, which is the underlying sequence set used to build Pfam version 35.0. Pfamseq 35 is based on UniProtKB Reference Proteomes (UniProt Consortium 2023) 2021_03 and contains 61 295 632 sequences and 23 910 108 270 residues, out of which 75.15% contain at least one match to one of the Pfam families. We run a single convolutional model from the ProtENN2 ensemble trained on Pfam version 35.0 against all the sequences in the Pfamseq 35 database and use the predictions to construct embeddings.

**Figure 2.**
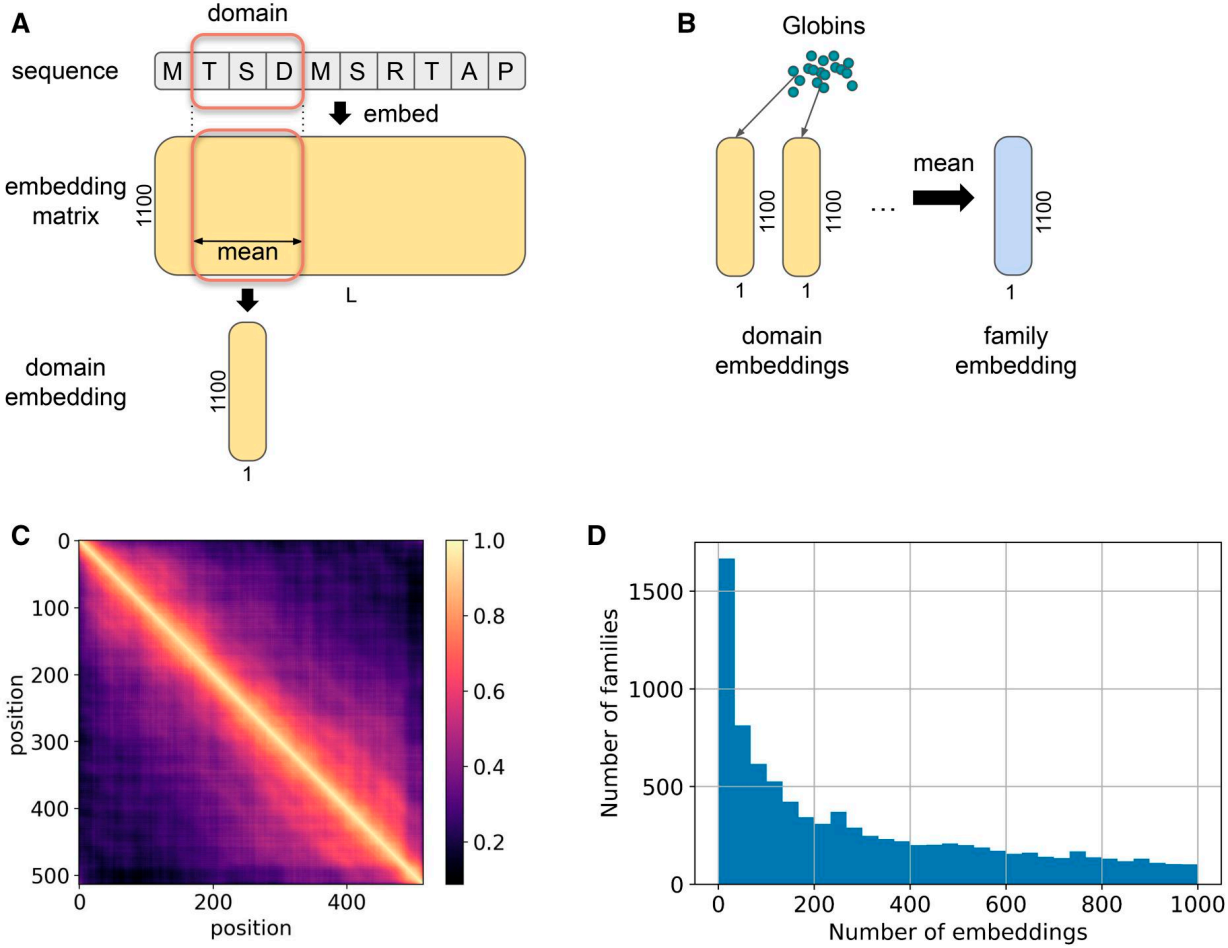
Method to calculate the domain embeddings and family embeddings. (A) For each sequence domain (of length L) in Pfamseq 35, the complete per-residue embedding (L by 1100) from ProtENN2 is calculated. Embedding vectors corresponding to positions within each domain called by the model are then averaged to create a domain embedding. (B) Domain embeddings are calculated across Pfamseq 35. Those called by this model to belong to each family are then averaged to yield a family embedding. (C) Heatmap of pairwise residue cosine similarities within sequence the drosophila G-protein coupled receptor Mth (UniProtKB: O97148), demonstrating the smoothness of convolutional embeddings along the sequence. (D) Distribution of the number of domain embeddings used to calculate family embeddings across Pfam families, only families with less than 1000 domain embeddings are shown here.

After running the model against Pfamseq 35, we obtain a set of predicted domains with corresponding coordinates and family labels. We require the predictions to be at least 20 amino acids long, where for each position, any predicted class label has a probability above the 0.025 threshold (Fig. 1B), following the thresholds used in Bileschi *et al.* (manuscript in preparation, https://zenodo.org/records/11187183). For each domain call made by the single ProtENN2 model, we take the per-residue embeddings from the last convolutional block and average across the span of the predicted domain (along the length axis, Fig. 2A). This produces a single 1100-dimensional vector for a specific instance of a domain that we call the ‘domain embedding’. The property of smoothness or continuity of convolutional embeddings in the embedding space, where adjacent residues have higher cosine similarity than the residues further apart in the sequence (Fig. 2C), allows us to expect that the averaged domain embedding will serve as a good domain representation. After that, for each family, we average the domain embeddings among all the instances of the family predicted by the model (Fig. 2B), this average of all the domain embeddings is called the ‘family embedding’. The distribution of the number of domain instances used for creating the family embeddings is shown in Fig. 2D for families containing <1000 domains.

**Figure 3.**
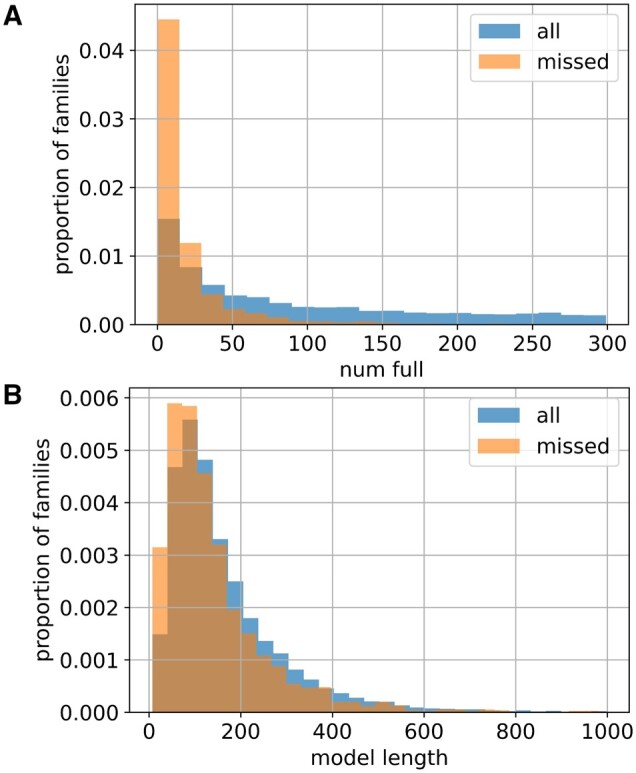
Distributions for families missed by ProtENN2 model and for all families in Pfam 35.0. (A) Distributions of numbers of sequences in FULL alignment. (B) Distributions of model lengths.

This process allows us to obtain embeddings for 17 317 families in Pfam version 35.0 (88.2% of all families). For some families, the model does not predict any occurrences of the domain, and as a result we are not able to construct embeddings for these families. Figure 3A shows that most of the missing families have relatively few sequences in the corresponding Pfam FULL alignment. We note that model length does not strongly affect the ability of the model to predict the family (Fig. 3B). It is also worth noting that the different models from the ProtENN2 ensemble miss different sets of protein families.

To measure the distance between two family embeddings, we develop a metric based on normalized cosine similarity. First, we calculate cosine similarity for each pair of family embeddings using the family-specific 1100-dimensional embeddings. Using this metric, we find that some families have many more close neighbours in the embedding space than others. Such cases include coiled coils, which have high pairwise cosine similarity but are not necessarily related, and unstructured regions, which represent highly disordered sequences and tend to have high similarity to other unstructured families. To account for this observation, we normalize the cosine similarity scores: we approximate the distribution of cosine similarities for each family by a Gaussian distribution and shift and rescale this distribution using the mean and variance of this distribution (1). In Equation (1), $\mu_{i}$ corresponds to the mean cosine similarity of all families to family ${\text{fam}}_{i}$, and $\sigma_{i}$ represents the standard deviation of the similarity distribution of all families to family ${\text{fam}}_{i}$.

(1) $$\text{normed}\_\text{sim}({\text{fam}}_{i},{\text{fam}}_{j})=\frac{\text{sim}({\text{fam}}_{i},{\text{fam}}_{j})-\mu_{i}}{\sigma_{i}}.$$

**Figure 4.**
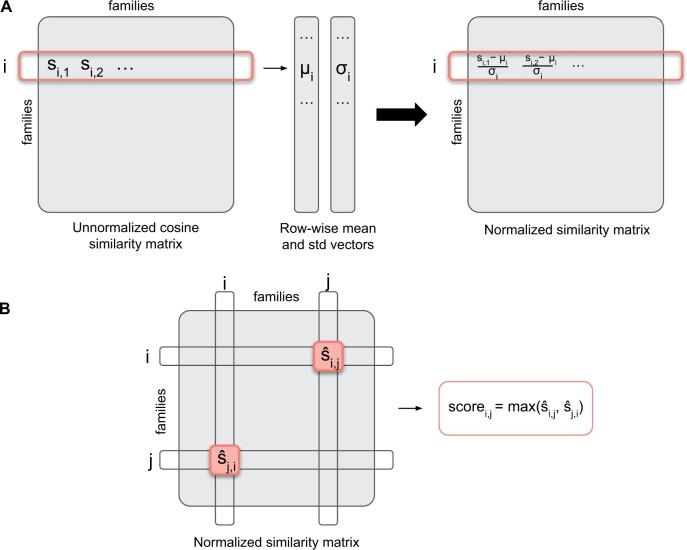
Illustrations of (A) row-wise similarity score normalization. (B) Final score aggregation method.

After we perform the normalization of the similarity score distributions for each family, for a pair of families ${\text{fam}}_{i}$, ${\text{fam}}_{j}$ we are left with two normalized cosine similarity values: the score for ${\text{fam}}_{i}$ with respect to ${\text{fam}}_{j}$, and vice versa, the score for ${\text{fam}}_{j}$ with respect to ${\text{fam}}_{j}$. As a result of the normalization, these scores are not symmetric, so to obtain a single score for each pair of families, we take the maximum of these two values (2). This approach means that for a strong prediction, at least one of the families in a pair needs to have a high normalized score against another. Empirically, we find that taking the maximum yields better results than averaging two scores, or taking the minimum, as measured using the metric defined below, see Supplementary Fig. S1 for details. Figure 4 illustrates the normalization and the final score aggregation process.

(2) $$\begin{array}{c} \text{score}({\text{fam}}_{i},{\text{fam}}_{j})=\max(\text{normed}\_\text{sim}({\text{fam}}_{i},{\text{fam}}_{j}),\\ \text{normed}\_\text{sim}({\text{fam}}_{j},{\text{fam}}_{i})). \end{array}$$

We use this score to determine which pairs of Pfam families our embedding-based similarity metric, which we call ProtCNNSim, predicts are homologous to each other.

### 2.3 Optimizing ProtCNNSim

To optimize our method, we ask whether ProtCNNSim can accurately identify families that belong to the same clan. We set this up as a binary classification problem where each pair of normalized family embeddings is classified as belonging to the same clan or not. We consider two evaluation approaches, which differ in their definition of a false positive prediction:

- a ‘conservative’ definition, where a prediction is called a false positive only if two families are in different clans
- a ‘liberal’ definition, where a prediction is called a false positive if two families are not in the same clan (e.g. at least one of them doesn’t have a clan label)

We use the conservative definition for evaluation, with results for the liberal definition provided in the supplementary materials (Supplementary Fig. S2).

To assess the binary classification performance of ProtCNNSim, a common approach is to consider the area under the receiver operating characteristic curve (ROC AUC), however, there are important performance nuances that are not captured by the ROC AUC such as the accuracy of the most confident predictions that the method makes. In this work, we care about the precision of the method for its most confident predictions, so our assessment focuses on the number of true positives at 100 false positives (TP@100FP). This metric characterizes the number of true positive predictions the model will make if we allow only 100 errors. This metric has the effect of encouraging the ROC curve to be steep in the high-confidence region. Since we are interested in the most confident predictions, we use the TP@100FP score for model selection and evaluation.

**Figure 5.**
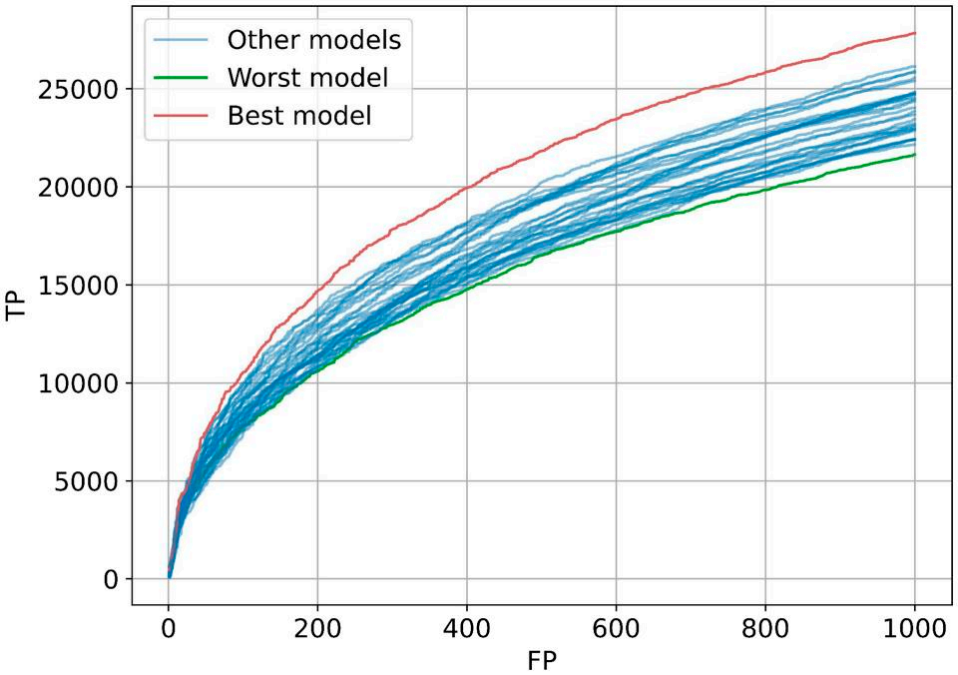
Sensitivity curves for members of the ProtENN2 ensemble show that there is a large difference between the performance of different ensemble elements, with the best and the worst models predicting 10 489 and 7577 true positives while making 100 false positive predictions.

To select the best-performing model from the ensemble, we randomly sample 1 000 000 sequences from Pfamseq 35 and run the whole pipeline from domain prediction to family embedding construction and evaluation for each of the 31 pre-trained models used in the ProtENN2 ensemble. Figure 5 shows the sensitivity curves for all 31 members of the ensemble. To construct the family embeddings, we selected the model which was able to identify the most (10 489) true family relationships while predicting exactly 100 false positive ones. We find that 100 false positives occur at a similarity score of 6.58, which is equivalent to a false discovery rate (FDR) of 0.94%.

**Figure 6.**
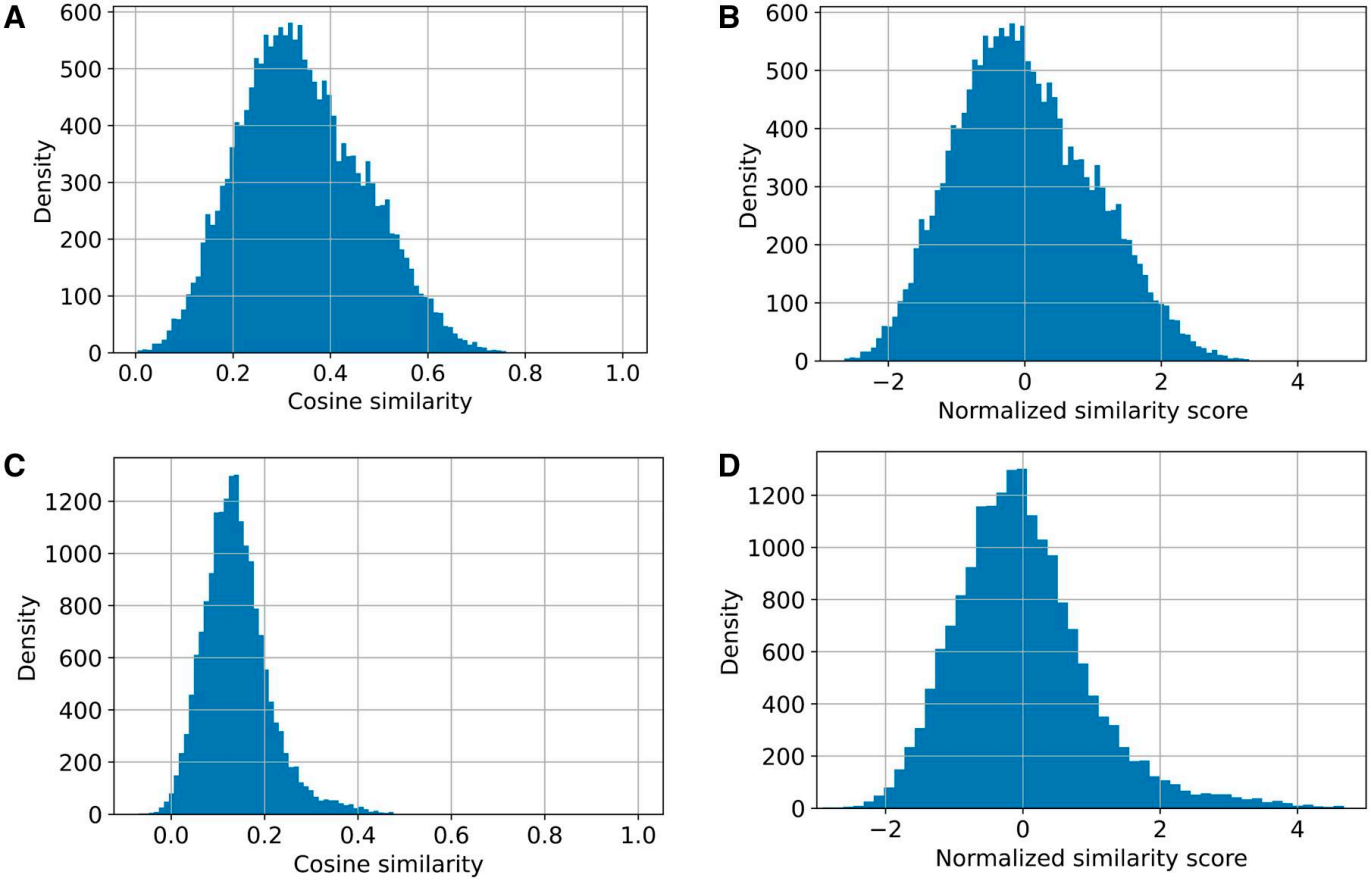
Examples of similarity distributions before and after normalization. The normalization places the similarity scores for the different families on a comparable scale. (A, B) Similarity scores for *TFIIS helical bundle-like domain* (Pfam:PF08711). (C, D) Similarity scores for *PhoLip_ATPase_C* (Pfam:PF16212).

**Figure 7.**
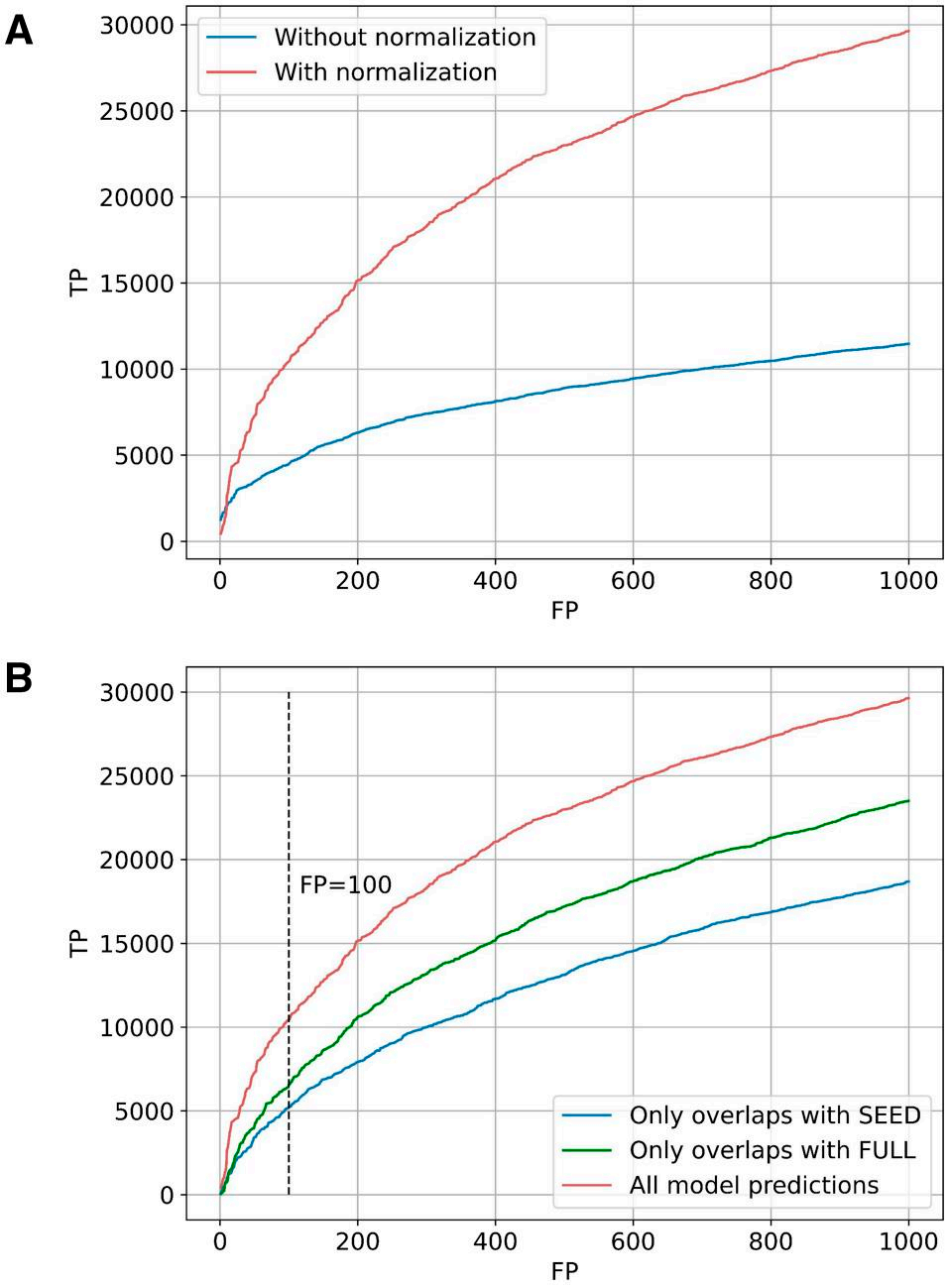
(A) Graph showing the classification results with and without normalization of the similarity matrix. (B) Sensitivity curves for the three different candidate methods used to construct the family embeddings.

In developing ProtCNNSim, we identified that certain groups of families showed biases in cosine similarity scores and thus we introduced a normalization procedure. The examples in Fig. 6 illustrate the effect of the similarity score normalization. For example, we note that before normalization, the ‘TFIIS helical bundle-like domain’ has higher similarity scores on average to other families than the ‘PhoLip_ATPase_C’ domain, but after the normalization, the scores are moved to the same scale and become comparable. Overall, we find that this normalization improves the performance of the method by a large margin (Fig. 7A).

To obtain domain predictions with which to construct the family embeddings, we run the best-performing model against protein sequences contained in the dataset ‘Pfamseq 35.0’. The precise boundaries of domains may differ between the Pfam alignments and what is called by this best-performing ProtENN2 model. We experimented with using only the model predictions that have a midpoint overlap (>50% residue overlap of either the model prediction with the Pfam-defined domain or vice versa) with domains from either SEED or FULL alignments of Pfam families, as well as using all the predictions made by the model. The results (Fig. 7B) show that the best-performing approach uses all available predictions from the model. Thus it appears that some significant power of the method comes from the additional predictions that ProtENN2 makes compared to the HMM-based methodology used by Pfam.

### 2.4 Comparison with existing methods

To compare the ability of ProtCNNSim to identify pairwise family relationships, we compare it to an existing method for family similarity prediction, the profile-profile comparison tool HHsearch from the HH-suite3 package (Steinegger *et al.* 2019). To create a fair comparison we move away from our Pfam-based assessment metric and use structural similarity between families as an independent benchmark. It is well-known that protein structures diverge more slowly than protein sequences (Chothia and Lesk 1986, Illergård *et al.* 2009). Therefore, we can use similarity in protein structure to identify when two proteins are homologous even when their sequence have diverged beyond the point they can be recognized as related. There is a long history of using structural similarity-based benchmarks to test sequence similarity algorithms (Park *et al.* 1997).

We use HHsearch with default parameters to calculate predicted similarity scores for all pairs of families in Pfam 35.0 and compare their predictions with our method. We note that the HHsearch method uses a database built using the SEED Pfam alignments, in contrast to ProtCNNSim, which was trained using Pfam FULL. To address this discrepancy, we tried to build databases using FULL Pfam alignments for both methods but we were unable to do this due to errors caused by memory constraints. Many Pfam families have very large FULL alignments with hundreds of thousands of sequences, with 1622 having more than 10 000 sequences and 82 families having more than 100 000 sequences in their FULL alignment (Supplementary Fig. S3).

To build structure similarity benchmarks, we use the predictions from TM-align algorithm, which provides structure similarity scores for protein pairs (Zhang and Skolnick 2005). To build a dataset of family models, for each family, we took the AlphaFold model for the first sequence in the SEED alignment which had an AlphaFold model (Jumper *et al.* 2021). For 18 217 families out of 19 632 families in Pfam 35 there was at least one SEED sequence with an AlphaFold model, which resulted in 93% coverage. For 17 198 of these models, they were inferred from the first sequence in the SEED alignment.

For these 18 217 AlphaFold models, we ran an all-versus-all TM-align comparison. We used the TM-align score normalized by the average length of two models as the final score, and defined matches as ground truth positives if they have a score equal or higher than *pos_th = *0.5. For defining the ground truth negative matches we used the threshold *neg_th *=* *0.17, which is described by Zhang and Skolnick (2005) as the score at which two random structures will match. Running TM-align and selecting the thresholds led to 463 104 positive predictions (0.14% of all pairs), which we call positives in structural comparison, and 34 959 773 negative predictions (10.6% of all pairs), which are used as negatives. We also tried to build sensitivity curves for other positive and negative thresholds for TM align scores, which can be found in supplementary materials (Supplementary Fig. S5). More stringent or relaxed thresholds illustrate more stringent or relaxed definitions of positive and negative matches. However, for the purpose of our assessment, we chose the thresholds suggested by the authors of TM align (Zhang and Skolnick 2005).

**Figure 8.**
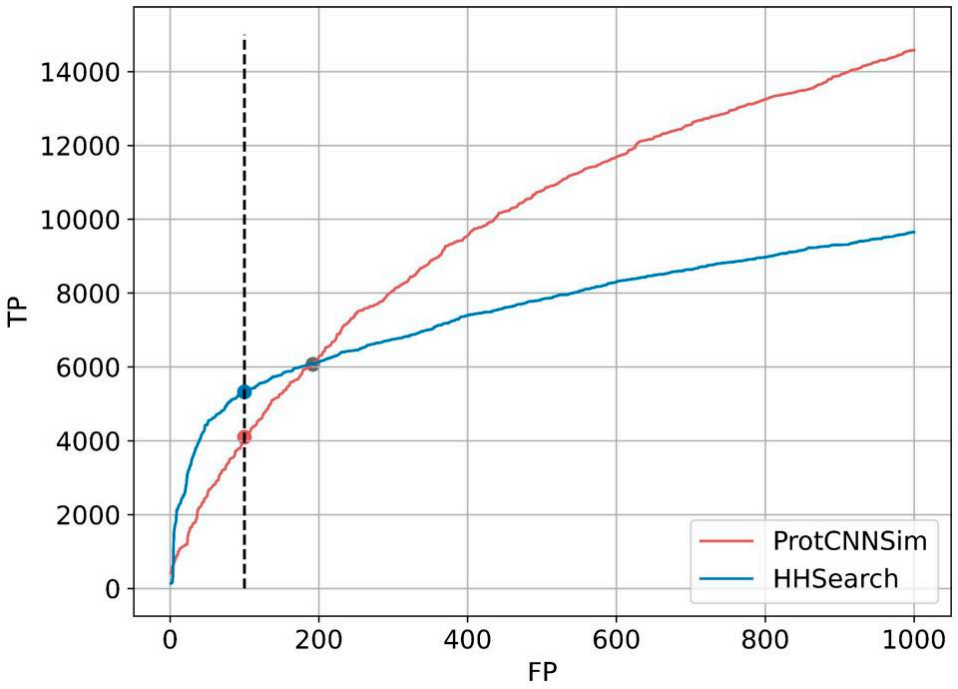
Sensitivity curves for ProtCNNSim and HHSearch on structure similarity benchmark defined by TM align scores. TM align matches with scores higher than *pos_th *=* *0.5 are considered ground truth positive for this evaluation. TM align matches with scores lower than *neg_th *=* *0.17 are considered ground truth negative. Dashed line shows true positive scores at 100 false positives for both methods (4096 for ProtCNNSim, 5324 for HHSearch). Between 192 and 193 false positives sensitivity curves intersect. ProtCNNSim has 6067 TP at 192 FP and 6104 TP at 193 FP, whereas HHSearch has 6085 TP at 192 TP and 6087 at 193 FP. ProtCNNSim score at 193 FP (crossover point) is 7.40.

To test ProtCNNSim’s ability to predict structural similarity, we used the aforementioned 463 104 predictions by TM-align as true positives for structural similarity and ran the analysis for ProtCNNSim and HHSearch predictions. To exclude false negatives, we removed from consideration 1415 Pfam families that didn’t have an AlphaFold model. Figure 8 shows that HHSearch outperforms ProtENN on this structure similarity prediction benchmark if we consider only highest scoring predictions (at 100 false positives HHSearch predicts 4096 true positives compared to 5324 true positives from ProtCNNSim), but as we allow more false positives, ProtCNNSim starts to provide more true positive predictions per false positive.

## 3 Results and discussion

### 3.1 Clustering with ProtCNNSim

**Figure 9.**
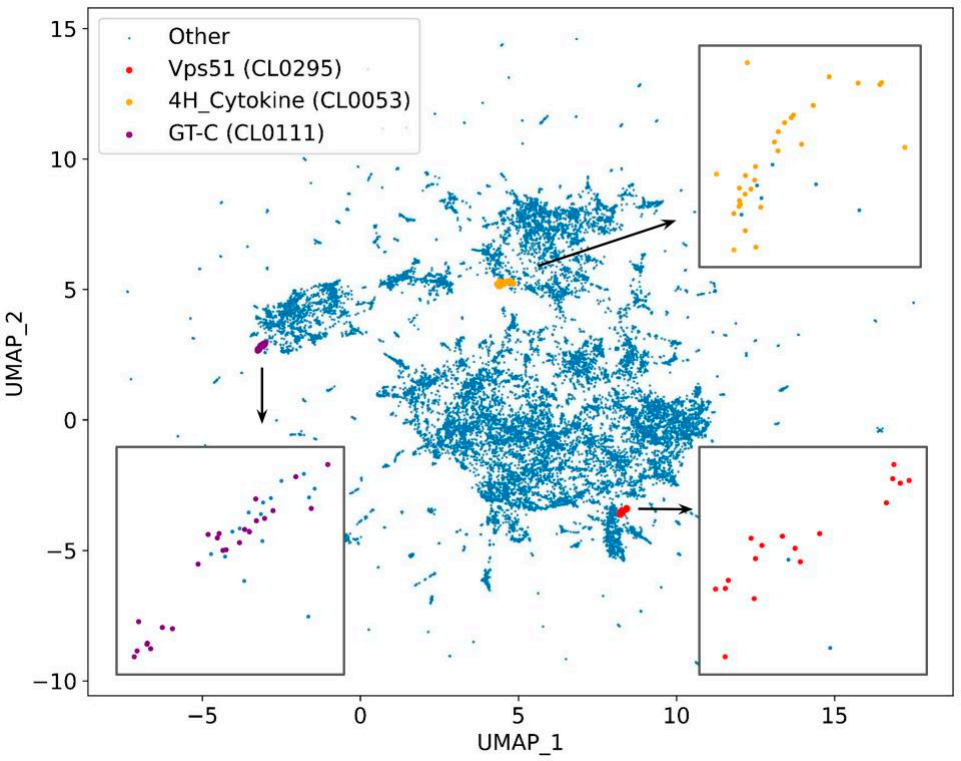
UMAP representation of all per-family embeddings. Each node represents a single Pfam family. Families found in three clans are highlighted: Vps51 (CL0295), 4H_Cytokine (CL0053) and GT-C (CL0111).

We were interested to see the overall clustering of the Pfam family embeddings, so we created a two-dimensional UMAP representation of the resulting family embeddings, which demonstrates the general tendency of embeddings to cluster (Fig. 9). The UMAP clustering takes the raw embeddings for each family as input and applies a cosine similarity scoring metric. Thus the UMAP clustering is most similar to the non-normalized scoring we have presented. We show some example clans highlighted on the UMAP clustering and can see that some clans demonstrate strong clustering among their members. Supplementary Fig. S4 shows more examples of clans visualized on the UMAP plot.

**Figure 10.**
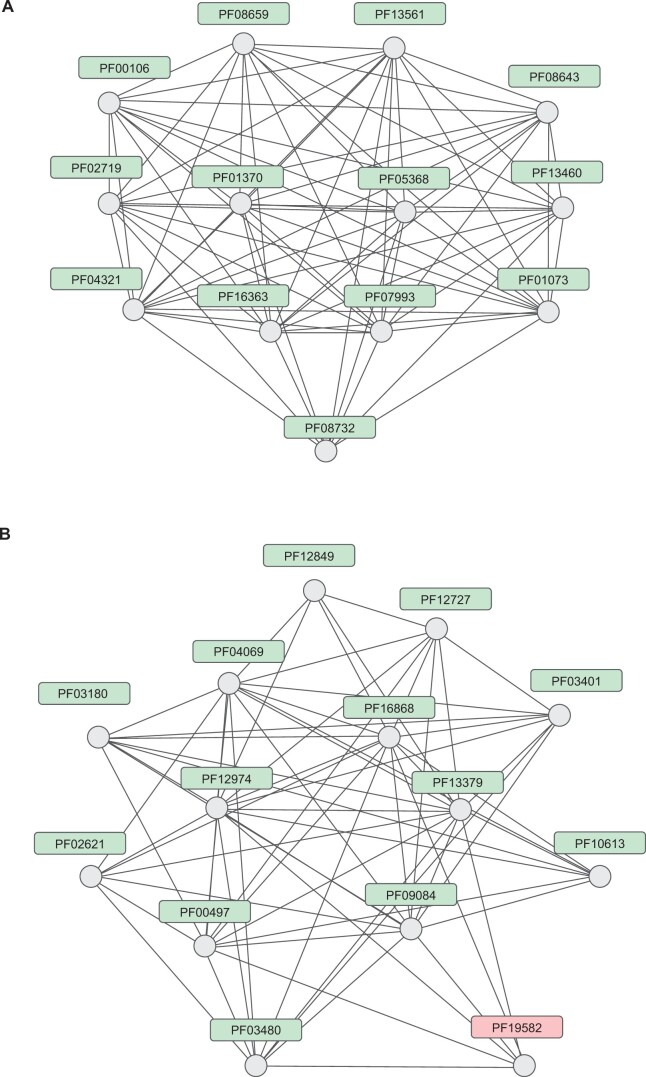
Examples of clusters found by the Leiden algorithm. The nodes represent Pfam families, the edges represent scores higher than 6.48. (A) Members of NADP_rossman clan (Pfam: CL0063) clustered together. (B) Members of PBPs clan (Pfam: CL0177) and solute-binding protein AdeT1/2 family (Pfam:PF19582) clustered together.

After the normalization procedure described above, which produces a similarity score for each embedding pair, we used these scores to build family clusters. To do this, we constructed a graph where the nodes represent families, and the edges represent pairwise similarities, using only those predictions that have similarity scores higher than 6.48, which corresponds to a 1% conservative FDR rate. For the resulting graph, the Leiden algorithm was used to find clusters, which can be interpreted as communities in the graph (Traag *et al.* 2019). We found that the majority of the bigger clusters represent parts of the existing clans (Fig. 10A). Some of the clusters, however, include families with no clan label, which suggests that these families might have a relationship to members of the cluster, which is absent from Pfam (Fig. 10B). Further we review some of these cases.

### 3.2 Analysis and interpretation of ProtCNNSim results: improving Pfam

We now move on to investigate the results in more detail to better understand our method’s strengths and weaknesses and to identify any interesting new biology that this approach uncovers.

To explore ProtCNNSim’s ability to discover novel family similarities and to further evaluate performance, we examined clusters of similar families created by the Leiden algorithm in more detail. The largest communities that contained new members were reviewed, and candidate members corroborated using additional evidence such as structural or sequence similarity. Most clusters that we obtained after running the Leiden clustering algorithm represent parts of existing clans. However, some clusters suggested adding new families to the clans, refining the clan labels, or creating new clans. Some examples are included in the section below.

#### 3.2.1 DUF6647 and Tox-MPTase5 belong to the peptidase clan MA

**Figure 11.**
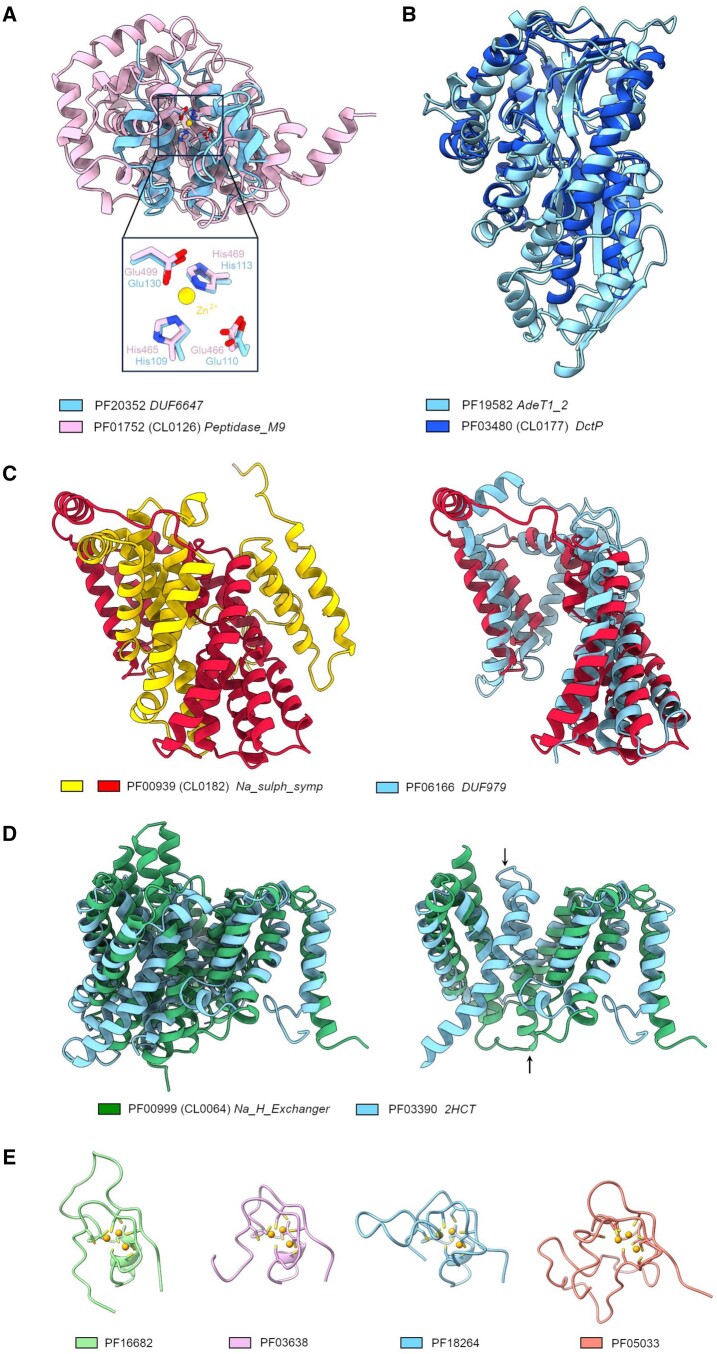
Examples of Pfam families added to clans as a result of the clustering. (A) Superposition of the AF2 model of DUF4157 domain-containing protein (UniProtKB:A0A2Z2P7G5, Pfam: PF20352) and peptidase domain of collagenase T (pdb:4ar9, Pfam:PF01752). Zn-coordinating residues and the catalytic glutamate are shown in sticks. (B) Superposition of the AF2 model of RND transporter (UniProtKB:A0A2U3MUG6, Pfam:PF19582) and TRAP dicarboxylate transporter, DctP subunit (pdb:4pdd, Pfam:PF03480). (C) Left: Structure of the DASS dicarboxylate transporter (pdb:6wtw, Pfam:PF00939). Right: superposition of the DASS dicarboxylate transporter structural repeat with the AF2 model of putative membrane protein (UniProtKB:A9GJV4, Pfam:PF06166). (D) Left: Superposition of sodium proton exchanger NHA2 (pdb:7b4l, Pfam:PF00999) and sodium-dependent citrate transporter KpCitS (pdb:5xar, Pfam:PF03390). Right: Superposition of individual structural repeats of NHA2 and KpCitS. The swapped helix 5 is indicated with an arrow. (E) CXC domains of MSL2 (pdb:4rkg, PF16682), LIN54 (pdb:5fd3, PF03638), EZH2 (pdb:4mi0, PF18264), Clr4 (pdb:1mvh, PF05033).

Peptidase clan MA (Pfam:CL0126) groups together a number of metallopeptidases that contain a highly conserved HEXXH sequence motif, which is part of a tripartite metal-coordinating site located within the enzyme active site (Rawlings and Barrett 1995). This sequence motif is also conserved in both the Tox-MPTase5 (Pfam:PF15641) and DUF6647 (Pfam:PF20352) families, which are not members of CL0126. ProtCNNSim finds that these families score highly against known members of the Peptidase MA clan. For example, DUF6647 has a score of 6.81 against DUF4157 (Pfam:PF13699), while Tox-MPTase5 has a score of 7.88 against DUF4157 (Pfam:PF13699), 7.77 against the WLM domain (Pfam:PF08325) and 7.69 against the SprT-like family (Pfam:PF10263) respectively, all of which belong to the Peptidase MA clan. In addition, these families contain either an invariant histidine or a glutamate residue which are located upstream of this motif and could serve as candidates for the third metal coordinating residue. It is likely that these two families contain the minimal structural features of this clan and belong to the group of the so-called ‘mini-zincins’, that are structurally exemplified by Acel_2062 protein (pdb:3e11) (Trame *et al.* 2014) (Fig. 11A).

#### 3.2.2 AdeT1_2 belongs to the periplasmic binding protein clan

Pfam clan CL0177 represents type II periplasmic binding proteins (PBPs) that are usually associated with one of the three bacterial transport systems and are divided into different types based on differences in their topology (Tam and Saier 1993, Fukami-Kobayashi *et al.* 1999). These proteins consist of two α/β domains with a ligand binding site located at the interface between these domains. A characteristic feature is the hinge consisting of two β-strands that connect these domains and function as a mediator of the ligand-induced changes to the relative orientation of the two domains. The AdeT1_2 family (Pfam:PF19582) gives significant matches to members of the PBP clan, for example, 7.44 against ABC transporter, phosphonate, periplasmic substrate-binding protein (Pfam:PF12974) and 7.19 against NMT1-like family (Pfam:PF13379) (Fig. 9B). This family groups together homologs of the uncharacterized solute-binding protein AdeT1/2. Members of this family have greater similarity to the tripartite ATP-independent periplasmic (TRAP) transporters from the PBP clan, in particular, to members of the DctP family (Pfam:PF03480). Besides the conserved topological features typical for type II PBPs, these proteins are likely to contain some extra structural components of the hinge, observed only in the TRAP PBPs, such as a long α-helix spanning the two domains and a highly conserved glycine residue found in a β-bulge at the hinge (Fig. 11B).

#### 3.2.3 DUF979 belongs to the ion transporter superfamily clan

The ion transporter (IT) superfamily clan (CL0182) groups together a number of integral membrane proteins primarily involved in the transport of ionic compounds and composed mainly of α-helices (Prakash *et al.* 2003). A typical feature of these proteins is the presence of two V-shaped internal structural repeats each consisting of seven α-helices with different lengths. These repeats are related by a two-fold symmetry and interlock to form the functional transporter protomer. DUF979 family (Pfam:PF06166) yields significant scores to members of the CL0182 clan, showing striking similarity to the divalent anion-sodium symporters family (Pfam:PF00939). In contrast to them, however, DUF979 proteins consist of a single repeat that likely represents their common ancestral fold. The presence of a single non-compact repeat also suggests that DUF979 proteins may function via homodimer formation (Fig. 11C). However, this conclusion was not supported by homodimer structure prediction using ColabFold (Mirdita *et al.* 2022). Investigation of the STRING networks (Szklarczyk *et al.* 2023) of DUF979 proteins suggested that the DUF969 (Pfam:PF06149) proteins, which are often closely located in operons, may serve as a potential interacting partner. The DUF979/DUF969 proteins were previously shown to reside in operons with proteins involved in 5-oxo-L-proline metabolism (Niehaus *et al.* 2017) and it was suggested they may play a role in its transport. An AlphaFold predictions using ColabFold of the DUF979 and DUF969 protein from *Clostridioides difficile* (UniProtKB:Q186N2 and UniProtKB:Q186N4) shows a confident prediction of their interaction with ipTM scores around 0.9 for the best model (see Supplementary Fig. S6). The individual AlphaFold structural models comparison of the DUF979 and DUF969 members revealed a significant similarity arguing for their common evolutionary past via possible gene duplication event (see Supplementary Fig. S6B). Hitherto known members of the CL0182 clan were shown to contain two structural repeats. The findings above present an interesting precedent of a probable ancestral heteromeric form from which the current families have originated and which is apparently intermediate between the presumed single repeat ancestor and the duplicate repeat descendant.

#### 3.2.4 2HCT belongs to the CPA/at transporter superfamily clan

In contrast to the families above, two members of the 2-HCT (Pfam:PF03390) family are structurally and functionally characterized. These are the sodium-dependent citrate symporters SeCitS (Wöhlert *et al.* 2015) and KpCitS (Kim *et al.* 2017). Members of the 2-HCT family are involved in the translocation of molecules containing a 2-hydroxycarboxylate motif, such as citrate, malate and lactate, across the membrane. These proteins are composed mainly of α-helices and contain two non-compact structural repeats that interlock to form the functional protomer. These structural repeats are related to the repeats observed in members of the CPA/AT transporter superfamily clan (Pfam:CL0064). They interlock in a similar manner and share a good structural similarity with the exception of the helix H5 that is swapped in other members of this superfamily (Fig. 11D).

#### 3.2.5 Merging and creating new clans

Several confident predictions suggest that there is a strong similarity between members of the clan CL0165 (Cache-like domain) and clan CL0183 (PAS domain clan). We note that in agreement with this observation made by the model, the ECOD structural classification places families from both of these clans into the same H-group, which is the level of homologous superfamily. Even though these two clans lack significant sequence similarity, their homology was previously described in Upadhyay *et al.* (2016). Taken together, these data strongly suggest that these two Pfam clans should be merged together.

Some clusters suggested relationships between proteins with intrinsic disorder or with limited secondary structures and these were used as a basis to create new clans. One example is clan CL0809 that now represents the CXC domain. This domain is a zinc-binding domain containing nine cysteine residues that coordinate three zinc ions. Despite having almost no secondary structures this domain has a compact fold that is built around an unusual Zn(3)Cys(9) cluster with three triangularly arranged zinc ions (Fig. 11E). Amongst the nine cysteines, six coordinate individually whereas three simultaneously bind two zinc ions. The CXC domain is found either in single or in tandem copies and has been implicated in DNA binding (Zheng *et al.* 2014, Marceau *et al.* 2016). This domain is found in a number of proteins, such as MSL2, Tesmin/TSO1, LIN54, HKMTs, etc., represented by distinct Pfam families that are now grouped in clan CL0809.

#### 3.2.6 Refining Pfam boundaries

**Figure 12.**
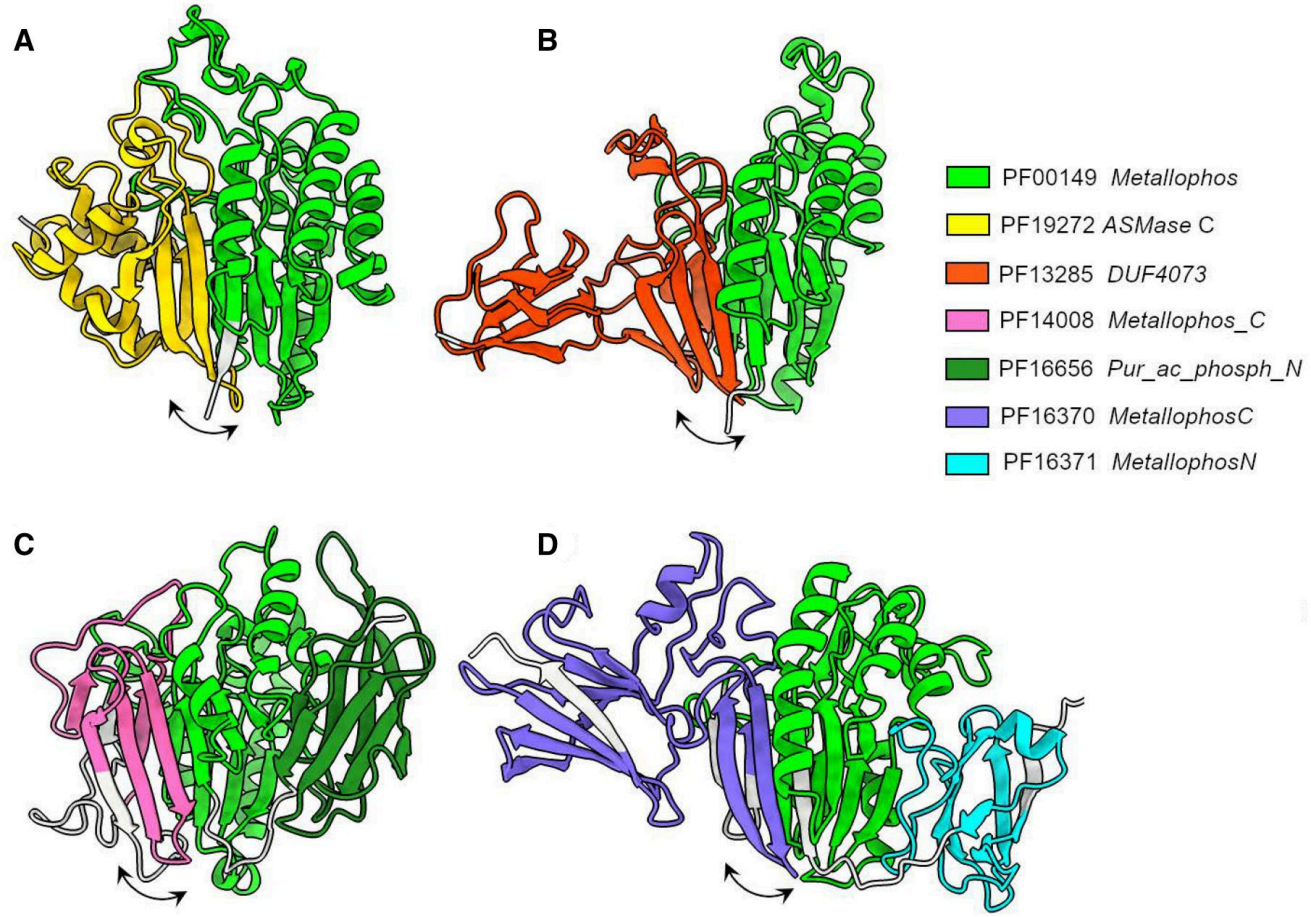
Examples of Pfam families containing regions with partial similarity of otherwise distinct domains. Regions in common in these domains are indicated with arrows on the AF2 models of: (A) *S.phosphodiesterase* acid like-3A (UniProtKB:U3KA47. (B) DUF4073 domain-containing protein (UniProtKB:C0ZKU3). (C) Purple acid phosphatase (UniProtKB:I3K264); (D) Uncharacterized protein (UniProtKB:R6ESA7).

A close inspection of some presumed false positive prediction clusters revealed that their constituent Pfam families contain regions with partial similarity which are included in otherwise distinct domains. For example, Pfam:PF00149, containing the catalytic core of a diverse range of phosphoesterases, is found in combination with distinct Pfam domains. The latter share a region in common comprising a small part of the catalytic domain (Fig. 12). Clusters of this kind were used for refining Pfam family definitions and for improving the current boundaries of domains.

### 3.3 Failure modes of ProtCNNSim

**Figure 13.**
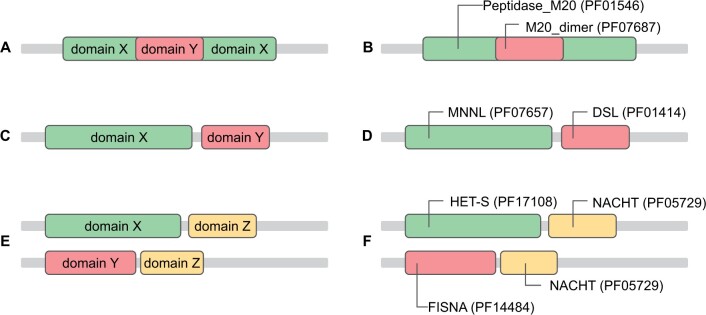
Schematic illustrations and examples of failure modes for ProtCNNSim. (A, B) Nested domain failure mode. Peptidase_M20 (Peptidase family M20/M25/M40, Pfam:PF01546) and M20_dimer (Peptidase dimerization domain, Pfam:PF07687) often appear as nested domains as shown in the figure, which can erroneously lead to a high ProtSimCNN similarity score (11.67). (C, D) Adjacent domain failure mode. Pfam families MNNL (N terminus of Notch ligand C2-like domain, Pfam:PF07657) and DSL (Delta serrate ligand, Pfam:PF01414) often exist next to each other in the same protein sequence, which erroneously leads to a high ProtCNNSim similarity score (8.19). (E, F) Shared adjacent domain failure mode: FISNA (Fish-specific NACHT associated domain, Pfam:PF14484) and HET-S (N-terminal small S protein of HET, non-prionic, Pfam accession:PF17108) have a shared adjacent domain NACHT (NACHT domain, Pfam:PF05729) which erroneously leads to a high ProtCNNSim similarity score (8.74).

Despite producing few errors among the most confident predictions, our method shows a range of failure modes that could be the subject of future improvements. In common with other methods ProtCNNSim is unable to distinguish between parts of the nested domain cases (Fig. 13A and B). Nested domains are a special type of domain architecture, where one of the domains appears inserted into a loop of another one. Unless care is taken errors can be easily made in similarity prediction. For the ProtENN2 model, this means that there’s a span of the protein sequence where two family labels are assigned to the amino acid residues at the same time (Fig. 13B), making the predicted embeddings similar, because per-residue embeddings for the overlapping positions will be used during the averaging stage to create per-family embeddings of both families. Thus, these two family embeddings will end up similar even if these two families are not related.

Another common failure mode is the erroneous prediction of coiled-coil containing families to have high similarity. Coiled coils are a known failure mode for sequence similarity prediction methods such as HMMER and often require additional curation effort (Mistry *et al.* 2013). In Pfam, families containing Coiled coils often have a higher family-specific threshold (gathering threshold) for determining family inclusion. The large majority of Pfam families have a per-domain gathering threshold of 27 bits, whereas many Coiled-coil families have per-domain thresholds in excess of 30 bits.

We found that some domains that commonly appear together in sequences are predicted to be similar by the ProtCNNSim method, even when they are clearly unrelated by structure (Fig. 13C and D). We believe this happens at least in part due to the use of convolutional models, where each residue’s context has a large effect on the model’s learned representation of the residue and adjacent residues. We observe that close-by residues are closer in the embedding space than those which are far away (Fig. 2C). It may be the case that other model architectures than CNNs are less prone to this kind of error. We note that such cases are prevalent among the most confident errors. We explored various co-occurrence metrics to try to correct for these cases, but this degraded overall performance and made the methodology more complex.

Another type of false positive prediction is the ‘shared adjacent domain’ case (Fig. 13E and F), where families X and Y do not appear together in sequences, but both of them are often found adjacent to the same domain Z. Since the context of the domain affects the embedding of the domain, if X and Z often appear together in protein sequences, as do Y and Z, then the presence of the shared context for X and Y might force them to be proximal in the embedding space even where there is no relationship between them. In the example case in Fig. 13F, the common NACHT domain brings the unrelated HET-S and FISNA domains close together in the embedding space.

## 4 Conclusions

Deep learning has already been demonstrated as a powerful method to extend sequence-based classifications of protein sequence domains. In this work we build on previous work to develop a new deep learning based approach that identifies relationships between known protein families from the Pfam database. Identification of family similarities is practically useful because it can suggest potential functions for uncharacterized families. These relationships are encoded in the Pfam database as a set of Clans which contain Pfam entries that are thought to have a common evolutionary origin. This classification often mirrors the superfamily level found in Protein structure classifications such as SCOP (Andreeva *et al.* 2020), CATH (Sillitoe *et al.* 2021), and ECOD (Schaeffer *et al.* 2019), with the advantage that even proteins without a structure could be analyzed and grouped.

Given the abundance of accurate protein structure models now available, one might also ask why not just use structure comparison to identify similarities between protein families? Lesk and Chothia showed in 1986 that structure diverges more slowly than sequence and so structure similarity would in principle remain after sequences had diverged beyond all recognition (Chothia and Lesk 1986). However, there are a number of reasons why sequence and structure methods remain complementary. Many proteins are disordered in solution and so structure comparison fails in these cases. Also, some domains are structurally diverse while retaining strong signals at the sequence level such as the cysteine patterns found in FeS-containing proteins. In this work, we find such an example in the CXC domain where we could identify several domains of known structure as being homologues despite a lack of strong structural similarity. Furthermore, structure comparison searches underperform, in terms of sensitivity and alignment accuracy, as the length of the target structures decreases. Often sequence comparison becomes more accurate than structure comparison for short domains particularly when having few or no secondary structures. Additionally, structure comparisons may fail in cases where homologous structures undergo significant conformational changes. Similarly, comparison of prediction models can be problematic as it is known that even the state-of-the-art structure prediction methods may not always correctly orient domains relative to each other.

We investigate to what extent the method is able to highlight novel discoveries such as families that are missing from Pfam clans and should be added as well as looking for completely novel clusterings that can form new Pfam clans. In this work we present several examples that provide new insights into the biology of existing Pfam families, some of which, representing functionally and structurally uncharacterized proteins. In the process of evaluation, there were valuable evolutionary findings and novel functional insights that could provide a basis for future analysis and experimental validation. Therefore, we believe the method is of practical value.

We have made great efforts to ensure that the method is as simple as possible with few tunable parameters. It seems likely that the approach we have developed could be applied to other per-residue domain level prediction methods. One attractive feature of the embedding-based techniques is that information from multiple sources can be combined in a relatively straightforward manner. For example, we can envisage a future method that combines information from both sequence embeddings and structure embeddings to provide the best of both worlds.

In this work, we demonstrate a methodology to combine per-residue sequence embeddings into a family embedding, then we show a methodology to apply to a large set of family embeddings from Pfam and score them in a pairwise fashion. We find that this methodology overall performs less well that state-of-the-art sequence comparison method HHsearch. However, we believe it is somewhat complementary to these methods and still helps to detect novel family relationships that could be used to improve the Pfam classification. Thus this work makes a useful methodological advance to detect protein family similarity using embeddings as well as making some novel biological discoveries.

## Supplementary Material

vbae132_Supplementary_Data

## Data Availability

The data and source code implementing in this article are available at github.com/iponamareva/ProtCNNSim, 10.5281/zenodo.10091909.
