# Supplementary material for: Investigation of protein family relationships with deep learning
Source: Bioinform Adv. 2024 Sep 18;4(1):vbae132. doi: 10.1093/bioadv/vbae132 (PMC11467057; doi:10.1093/bioadv/vbae132)
Supplement: vbae132_Supplementary_Data [file vbae132_supplementary_data.pdf]

Supplementary materials

Investigation of protein family relationships with Deep Learning

Irina Ponamareva[1,3*], Antonina Andreeva[1], Max Bileschi[2], Lucy Colwell[2,3] and Alex Bateman[1]

[1]European Molecular Biology Laboratory, European Bioinformatics Institute (EMBL-EBI), Wellcome Genome Campus, Hinxton, Cambridgeshire CB10 1SD, UK, [2]Google Research, Cambridge, MA 02142, USA, [3]Department of Chemistry, University of Cambridge, Cambridge CB2 1EW, UK

*To whom correspondence should be addressed.

Contact: ip381@cam.ac.uk

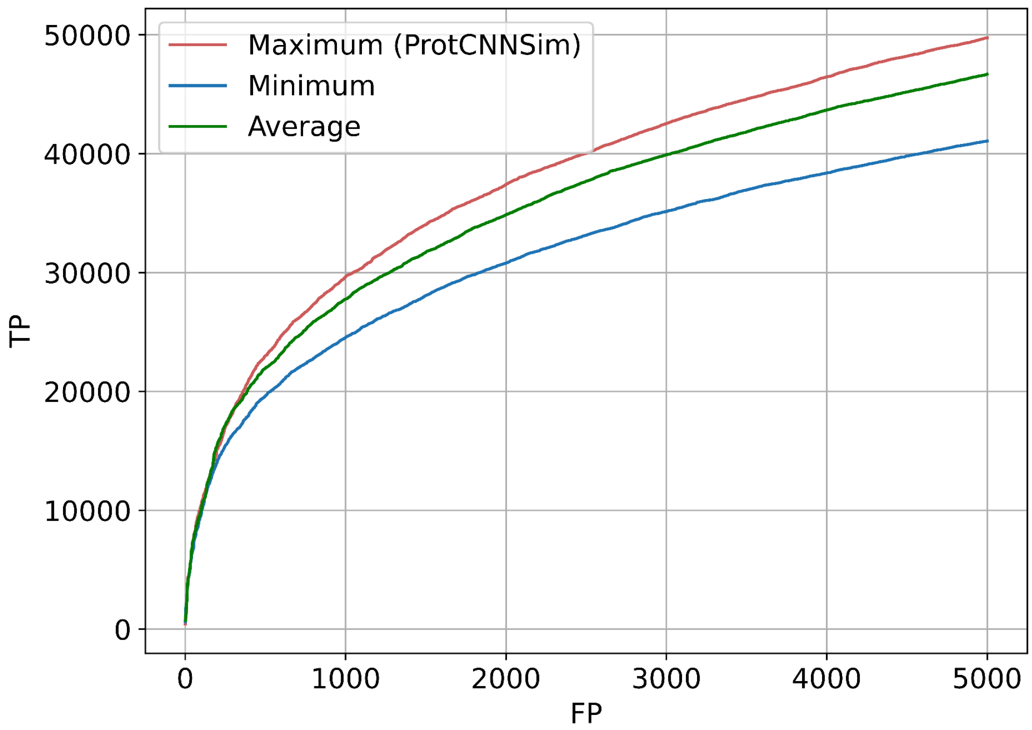

Figure S1. Sensitivity curves showing a comparison of taking the maximum, minimum and average of two similarity scores in ProtCNNSim method.

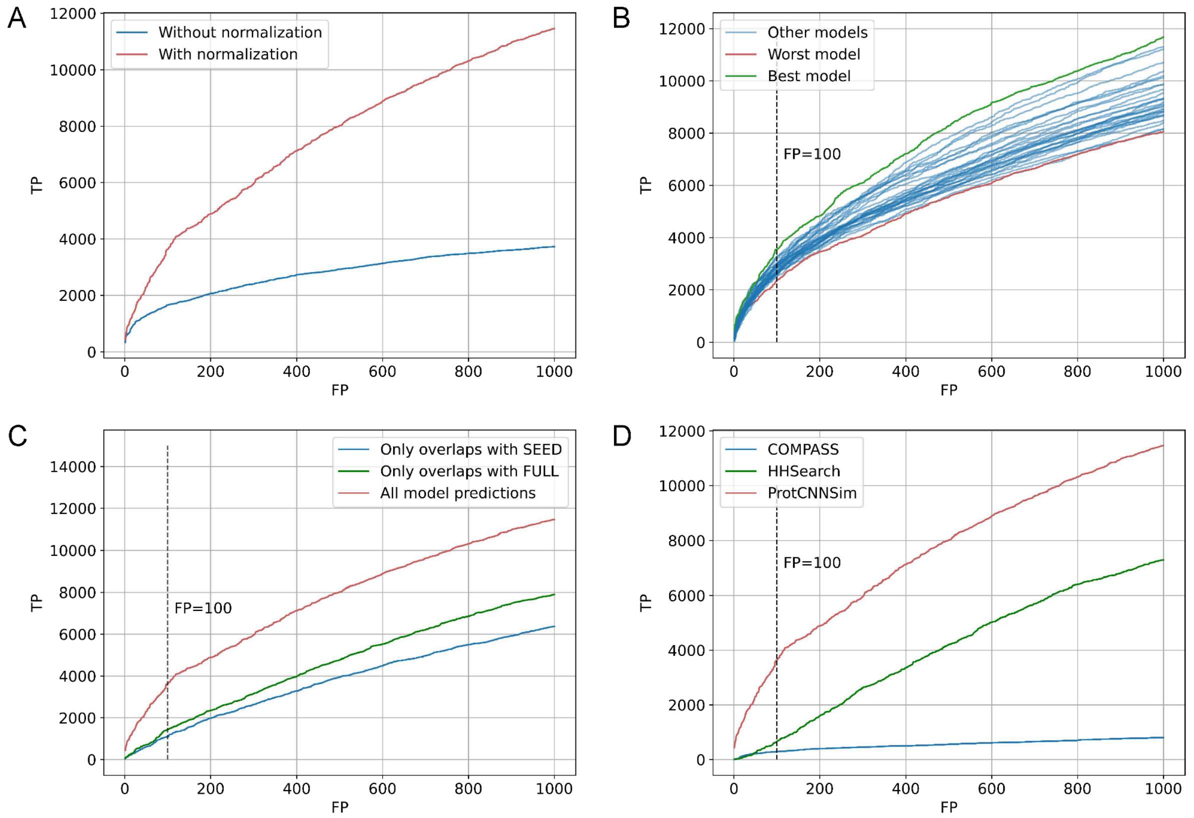

Figure S2. Sensitivity curves for the liberal definition of a false positive. (A) Classification results with and without normalisation of the similarity matrix, similar to Figure 7A. (B) Ensemble members, similar to Figure 5. (C) Three different methods used for constructing the family embeddings, similar to Figure 7B. (D) Family similarity prediction results using ProtENN, HHsearch, and COMPASS, similar to Figures 9A, 9B.

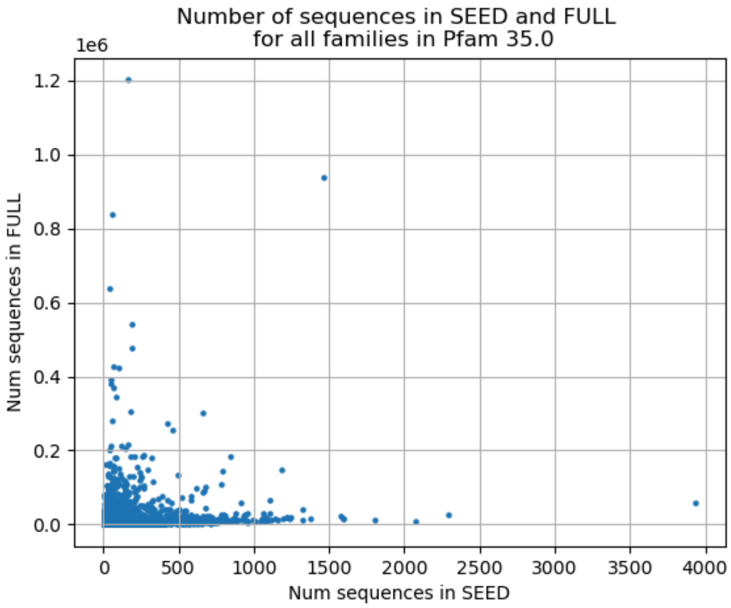

Figure S3. Number of sequences that are included in SEED and FULL alignments for all families in Pfam version 35.0.

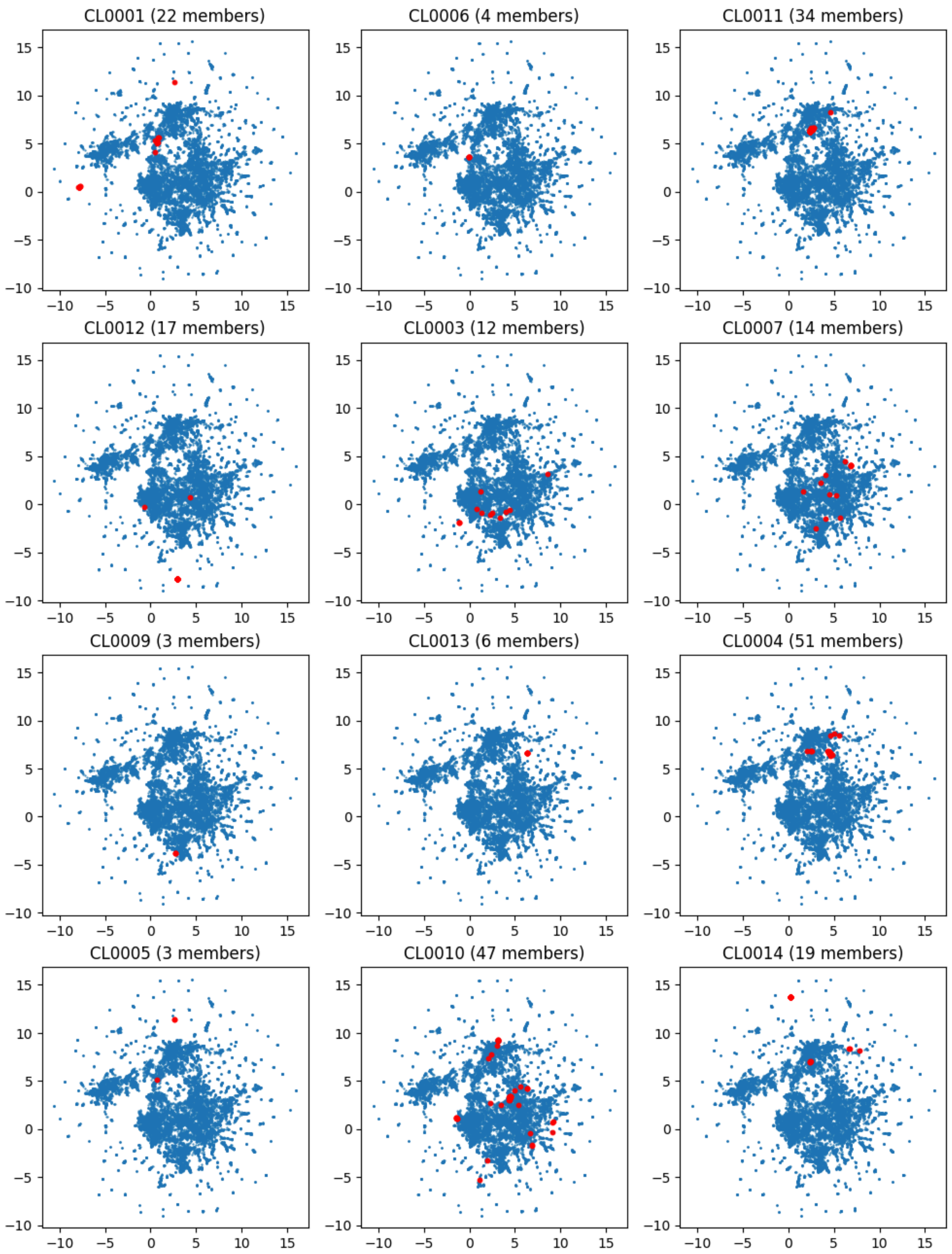

Figure S4. UMAP representation of family embeddings with particular clans highlighted.

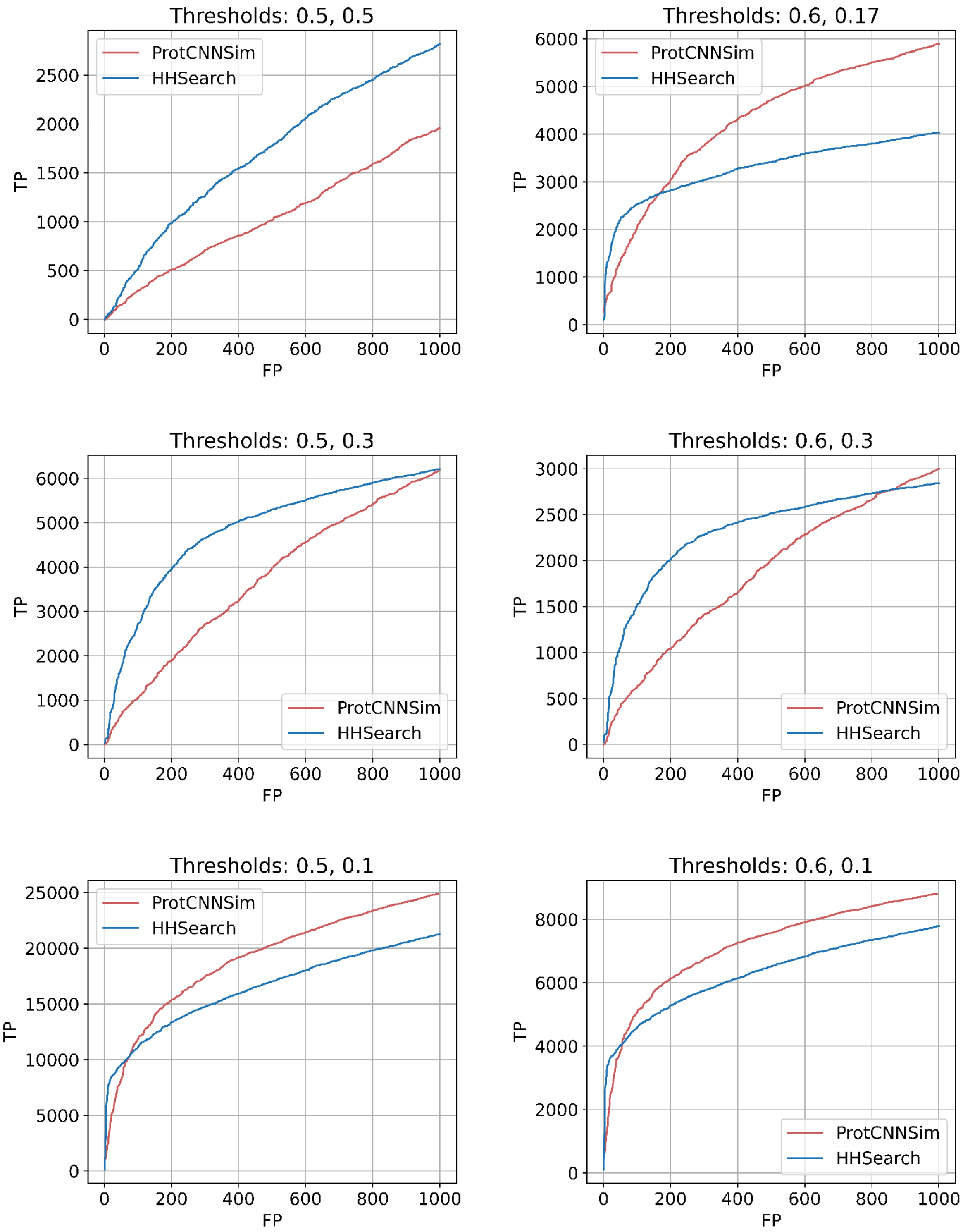

Figure S5. Sensitivity curves for ProtCNNSim and HHSearch on structure similarity benchmark defined by TM align scores with different positive and negative thresholds for TM align scores. TM align matches are defined as ground truth positives, if they have a score equal or higher than *pos_th* (left value in the title), and ground truth negatives, if they score below neg_th (right value in the title).

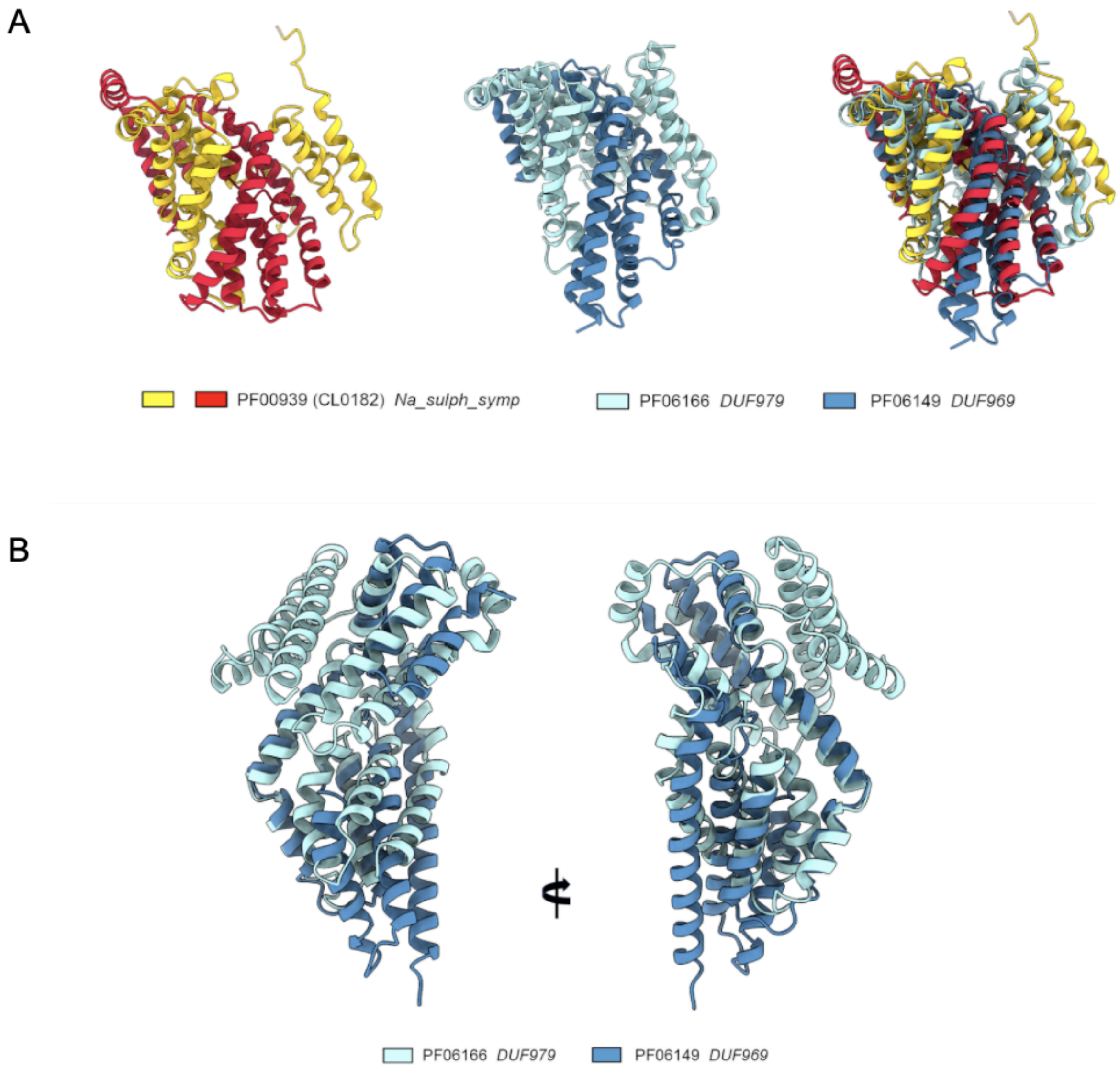

Figure S6. (A) Side-by-side: the structure of DASS dicarboxylate transporter (pdb:6wtw, Pfam:PF00939); AlphaFold prediction model of the complex of putative membrane proteins members of DUF979 and DUF969 (UniProtKB: Q186N2 and Q186N4, shown in dark and light blue) and their structure superposition. The internal structural repeats of DASS dicarboxylate transporter are shown in yellow and red. (B) Structure comparison of the individual AlphaFold prediction models of the DUF979 and DUF969 putative membrane proteins (UniProtKB: Q186N2 and Q186N4). The superposed models are shown in two different views.
